# Genome-wide identification and analysis of the Universal Stress Protein (USP) family in pepper (*Capsicum annuum* L.)

**DOI:** 10.3389/fpls.2026.1957880

**Published:** 2026-09-16

**Authors:** Li Chen, Zhixue Dong, Xuewei Wang, Zhuqing Zhang, Sha Yang, Xuefeng Li, Xuexiao Zou, Chengliang Liang, Wenchao Chen

**Affiliations:** 1 Institute of Vegetable Research, Hunan Academy of Agricultural Science, Changsha, China; 2 Yuelushan Laboratory, Changsha, China

**Keywords:** *Capsicum annuum* (pepper), *CaUSP* gene family, genome- wide analysis, pollen fertility, reproductive development

## Abstract

**Introduction:**

Members of the Universal Stress Protein (USP) family are crucial for plant abiotic stress responses and growth regulation.

**Methods:**

In this study, we identified 30 *CaUSP* genes in the pepper genome. These genes were distributed across all 12 chromosomes and phylogenetically classified into eight distinct subgroups. Collinearity analysis revealed evolutionary relationships between the USP families of pepper and *Arabidopsis*. Cis-acting element analysis indicated that *CaUSP* gene promoters harbour multiple stress-, hormone-, and development-related motifs. Expression profiling showed that *CaUSP19* is highly expressed in floral organs, with a marked increase during the late bud development stage, suggesting a potential role in reproductive development. Using virus-induced gene silencing (VIGS), we demonstrated that knockdown of *CaUSP19* significantly reduced pollen viability and germination.

**Results and Discussion:**

Collectively, this study provides a systematic genome-wide overview of the pepper *CaUSP* family and reveals a novel role for *CaUSP19* in regulating pollen viability, thereby laying a foundation for further investigation of USP functions in plant reproduction.

## 1 Introduction

Biotic and abiotic stresses are major constraints on the yield and quality of economic crops. Plants perceive stress signals through receptors, which activate stress-responsive genes and transduce external signals to downstream effectors, enabling adaptation to adverse conditions (Dan et al., 2023; Wang et al., 2024). Universal Stress Proteins (USPs) constitute a class of small proteins defined by the presence of one or more conserved USP domains (Sylvain et al., 2011). Originally identified in *Escherichia coli* as responders to multiple stressors (VanBogelen et al., 1990), USPs are markedly induced in bacteria under diverse stress conditions, such as extreme temperatures, nutrient limitation, oxidative stress, and ion toxicity (Kristian et al., 2003). Subsequent studies have revealed their widespread distribution across archaea, fungi, protists, plants, and animals (Kristian et al., 2003; Shatil et al., 2021; Xiaoyue et al., 2021), linking them to nearly all types of stress responses and to resistance against both abiotic and biotic challenges (Ralf et al., 2008; Rachid et al., 2012; Shatil et al., 2021; Kieu Anh Thi et al., 2022; Sirui et al., 2025).

In plants, USPs were first described in rice (Margret et al., 2002). Higher plant USPs display considerable structural and functional diversity: in addition to the core USP domain, some members possess kinase, U-box, zinc finger, or other functional domains (Young Jun et al., 2016). In *Arabidopsis*, *AtUSP* acts as a molecular chaperone under heat or cold stress, maintaining the activity of target proteins or RNAs and thereby enhancing stress tolerance (Hunseung et al., 2012; Silvia et al., 2015; Young Jun et al., 2016; Sarah Mae Boyles et al., 2017). In tobacco, USPs recognize phosphorylation signals triggered by salt and osmotic stress to promote downstream gene expression and osmolyte accumulation (Pushpika et al., 2016). Systematic bioinformatics analyses across 13 *Solanaceae* materials have identified 438 USP members, including cultivated tomato and its wild relatives; the stress-tolerant wild species *S. pennellii* harbors five unique USPs (Ruiqiang et al., 2025). In *Populus trichocarpa*, 46 *PtrUSP* genes were identified, with their promoters enriched in cis-acting elements related to hormone and stress responses (Jian et al., 2023). In *Gossypium arboreum*, two USPs are strongly induced by drought and help regulate cellular water content (Maqbool et al., 2009). Similarly, in potato, *StUSP13*, *StUSP14*, *StUSP15*, and *StUSP41* have been proposed as key candidates involved in stress responses (Tianshuai et al., 2024). while in grapevine, *VyUSPA3* positively regulates drought resistance in Chinese wild grape (*Vitis yeshanensis*) (Xiaoyue et al., 2022). A genome-wide survey in *Vaccinium* identified 72 *VcUSP* genes, of which 21 and 7 were differentially expressed under UV-B radiation and exogenous ABA treatment, respectively (Yan et al., 2023).

Beyond stress adaptation, USPs participate in various aspects of plant growth and development. In *Arabidopsis*, the USP encoded by *At3g58450* promotes seed germination through an abscisic acid-independent pathway (Gorshkova et al., 2018). While another USP (*At3g53990*) regulates the circadian rhythms of central oscillator genes (Kieu Anh Thi et al., 2022). GRUSP, a member of the USP family, is involved in gibberellin-dependent flowering induction (Gorshkova et al., 2018). In sweet potato, the nucleus-localized *IbUSP17* is highly expressed in high-starch varieties and during storage root expansion; its overexpression significantly increases starch content without yield penalty (Jian et al., 2023).

Pepper (*Capsicum annuum* L.) is an economically important vegetable and a key member of the Solanaceae family, which also includes tomato, tobacco, potato, and petunia (Seungill et al., 2014). Pepper is a diploid species with a somatic chromosome number of 2n = 2x = 24, although karyotype analyses have revealed variations in chromosome morphology and total haploid chromatin length among different cultivated varieties (Cheema and Pant, 2013). Although *USP* genes have been studied in several solanaceous crops, they remain largely unexplored in pepper, especially in the context of reproductive development. In this study, we identified 30 *CaUSP* genes in the pepper genome and systematically characterized their chromosomal distribution, phylogenetic relationships, promoter cis-elements, and expression profiles. We found that *CaUSP19* is highly expressed during late bud development, and its silencing severely impairs pollen viability and fertility. These results expand the functional understanding of the *CaUSP* family and provide a foundation for elucidating the mechanisms by which USPs regulate fertility in plants.

## 2 Results

### 2.1 Identification of the CaUSP family

We identified 30 *CaUSP* genes in the pepper genome (**Supplementary Table S1**). The encoded proteins vary in length from 145 to 849 amino acids, with predicted molecular weights ranging from 16.05 to 95.15 kDa and theoretical isoelectric points (pI) between 4.72 and 9.80 (**Supplementary Table S2**). Subcellular localization prediction indicated that the 30 CaUSP proteins were mainly localized in the nucleus (18 members), cytoplasm (9 members), and chloroplast (3 members), with the nucleus being the most frequently predicted localization (**Supplementary Table S2**). These predictions provide preliminary information on the potential subcellular distribution of CaUSP proteins and require further experimental validation. Using the published pepper genome sequence (BioProject accession no. PRJCA025503; the raw sequencing data have been deposited at the Genome Sequence Archive (https://ngdc.cncb.ac.cn/gsa/), we mapped all *CaUSP* genes to their chromosomal locations. These genes are distributed across all 12 chromosomes (**Figure 1A**). Based on their chromosomal order, they were designated *CaUSP1* through *CaUSP30* (**Supplementary Table S3**).

**Figure 1:**
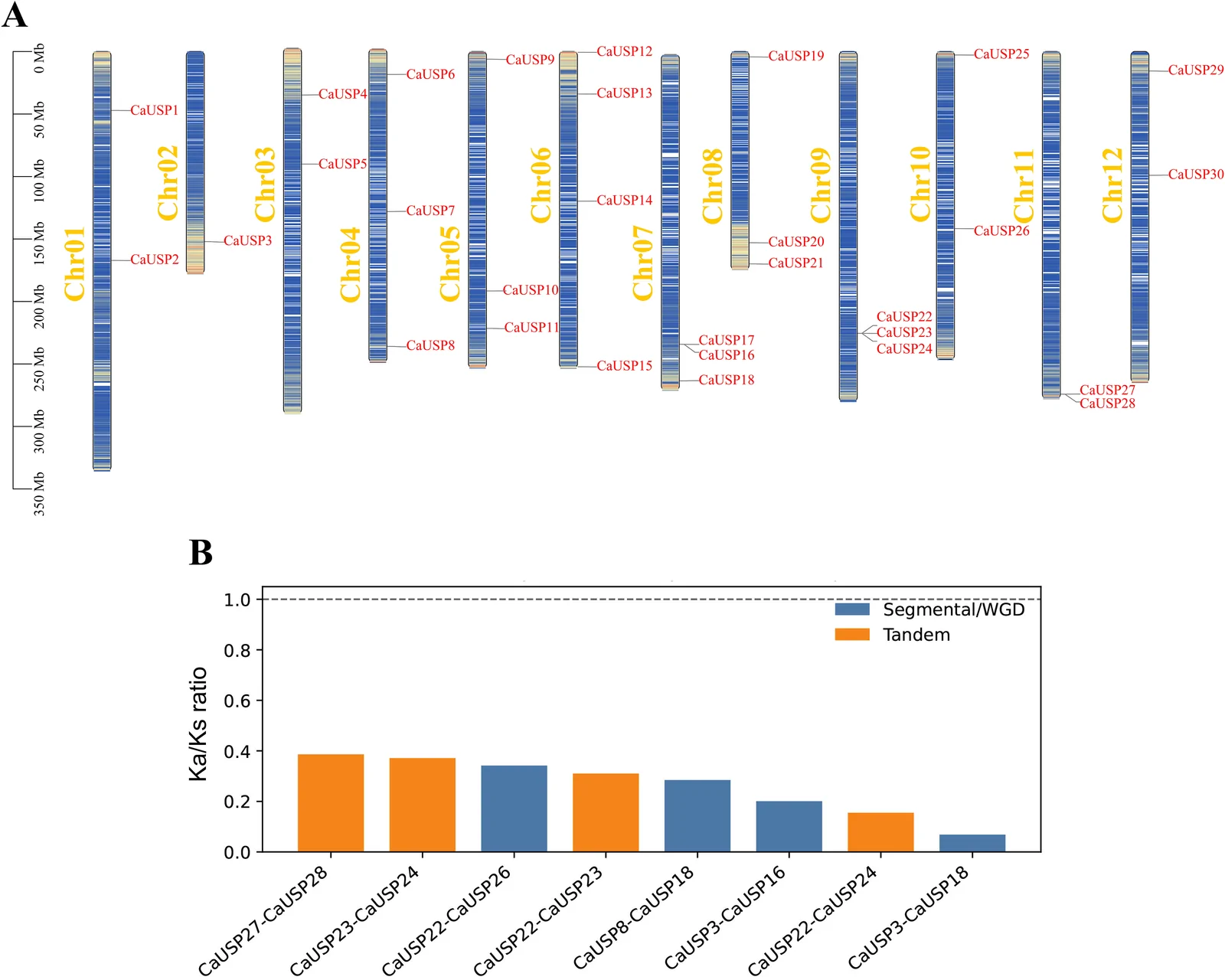
Chromosomal distribution and selective pressure analysis of duplicated CaUSP genes. **(A)** Chromosomal distribution of CaUSP genes across the 12 chromosomes of pepper. The y-axis indicates chromosome length (Mb). **(B)** Ka/Ks ratios of eight duplicated CaUSP gene pairs, including three tandem duplicated pairs and five segmental/WGD duplicated pairs. The x-axis indicates the duplicated gene pairs, and the y-axis represents the Ka/Ks ratio. Blue and orange bars represent segmental/WGD and tandem duplicated gene pairs, respectively. The dashed horizontal line indicates Ka/Ks = 1.

To investigate the evolutionary constraints acting on duplicated CaUSP genes, Ka/Ks ratios were calculated for eight duplicated gene pairs. The Ka/Ks values ranged from approximately 0.07 to 0.39, and all were below 1 (**Figure 1B**). This pattern was consistently observed for gene pairs derived from both tandem and segmental/WGD duplication events, indicating that duplicated CaUSP genes have predominantly undergone purifying selection. These findings suggest that evolutionary constraints have contributed to the conservation of CaUSP genes following duplication.

### 2.2 Phylogenetic analysis and classification of CaUSP family members

To investigate the evolutionary relationships of CaUSP proteins, a maximum-likelihood phylogenetic tree was constructed using USP proteins from *Arabidopsis thaliana* and *Oryza sativa* as references. Based on the tree topology and nodal support values, the CaUSP proteins were classified into five major groups (Groups I–V), whereas members with isolated or unresolved phylogenetic positions were provisionally assigned to Group VI (**Figure 2**). Group I contained nine members (CaUSP19, CaUSP1, CaUSP11, CaUSP5, CaUSP13, CaUSP29, CaUSP30, CaUSP10, and CaUSP14); Group II contained six (CaUSP25, CaUSP18, CaUSP8, CaUSP3, CaUSP17, and CaUSP16); Group III contained six (CaUSP26, CaUSP23, CaUSP24, CaUSP22, CaUSP2, and CaUSP9); Group IV contained two (CaUSP15 and CaUSP12); Group V contained CaUSP27 and CaUSP28; and Group VI contained the remaining members with isolated or unresolved positions, including CaUSP4, CaUSP6, CaUSP7, CaUSP20, and CaUSP21.

**Figure 2:**
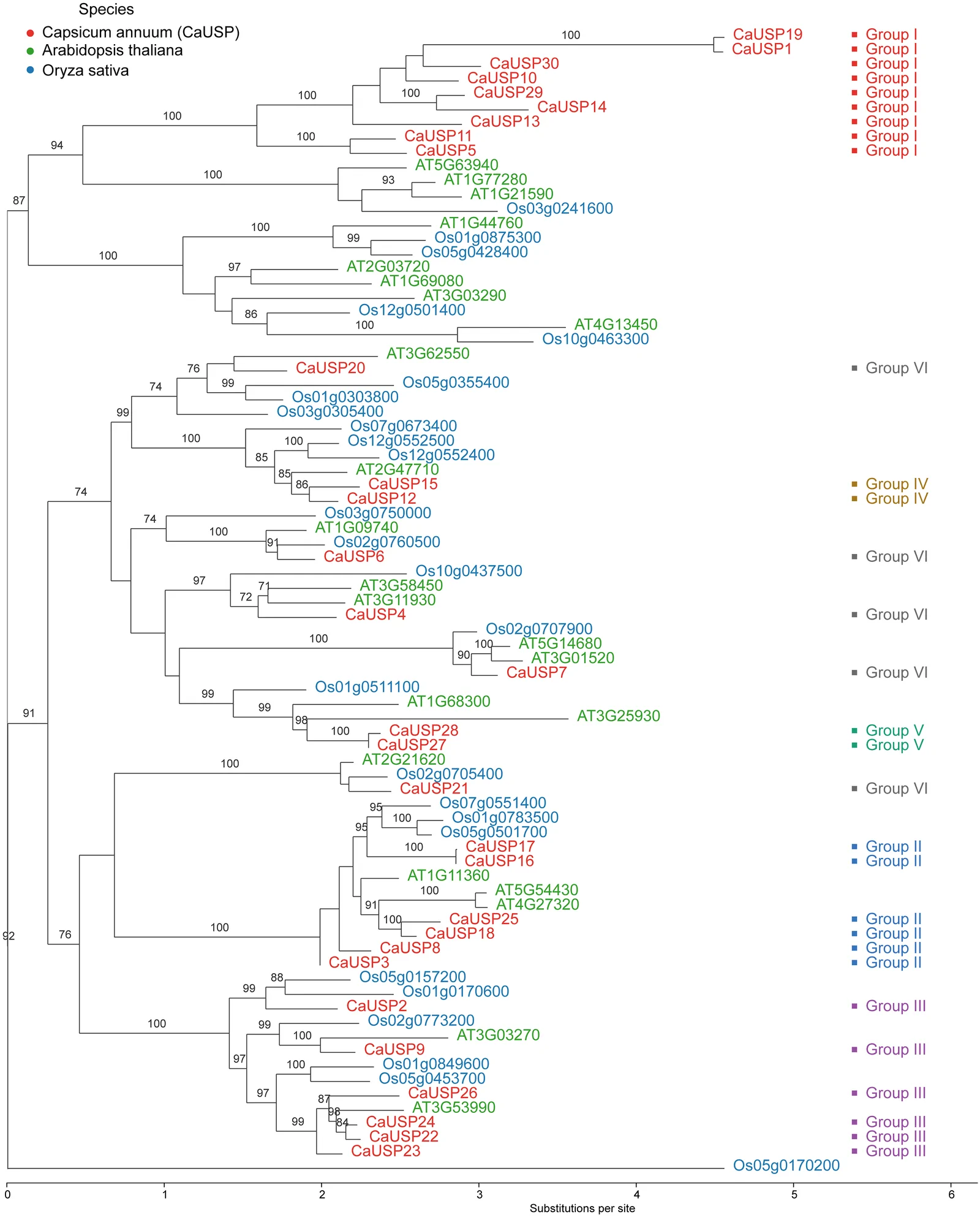
Phylogenetic analysis of USP proteins from pepper (*C. annuum*), *Arabidopsis* (*A. thaliana*), and rice (*O. sativa*).

### 2.3 Structural and motif analysis of the CaUSP family

Analysis of gene structures showed that the number of introns in *CaUSP* genes ranges from zero to nine, with *CaUSP10* containing the maximum (nine introns). We identified ten conserved motifs across the *CaUSP* family. Individual members harbored three to nine motifs, and proteins within the same phylogenetic subgroup shared highly similar motif compositions. Notably, Motif 1, which constitutes the core of the conserved USP domain, was present in all members. Unique motifs (Motifs 4–8) were found in a few subgroup-specific members, implying potential functional diversification among these genes (**Figure 3**). Domain architecture analysis showed that all 30 CaUSP proteins were predicted to contain a single USP domain, with no tandem USP domains identified. Several longer CaUSP proteins were predicted to contain additional domains, including protein kinase domains in CaUSP5, CaUSP10, CaUSP11, CaUSP13, CaUSP29, and CaUSP30, and U-box domains in CaUSP5 and CaUSP11. Based on the Walker-type ATP-binding signature, 13 CaUSPs were classified as ATP-binding-type candidates and 17 as non-ATP-binding-type candidates. Notably, this signature was not detected in CaUSP19, suggesting that CaUSP19 may represent a non-ATP-binding-type candidate (**Supplementary Table S4**).

**Figure 3:**
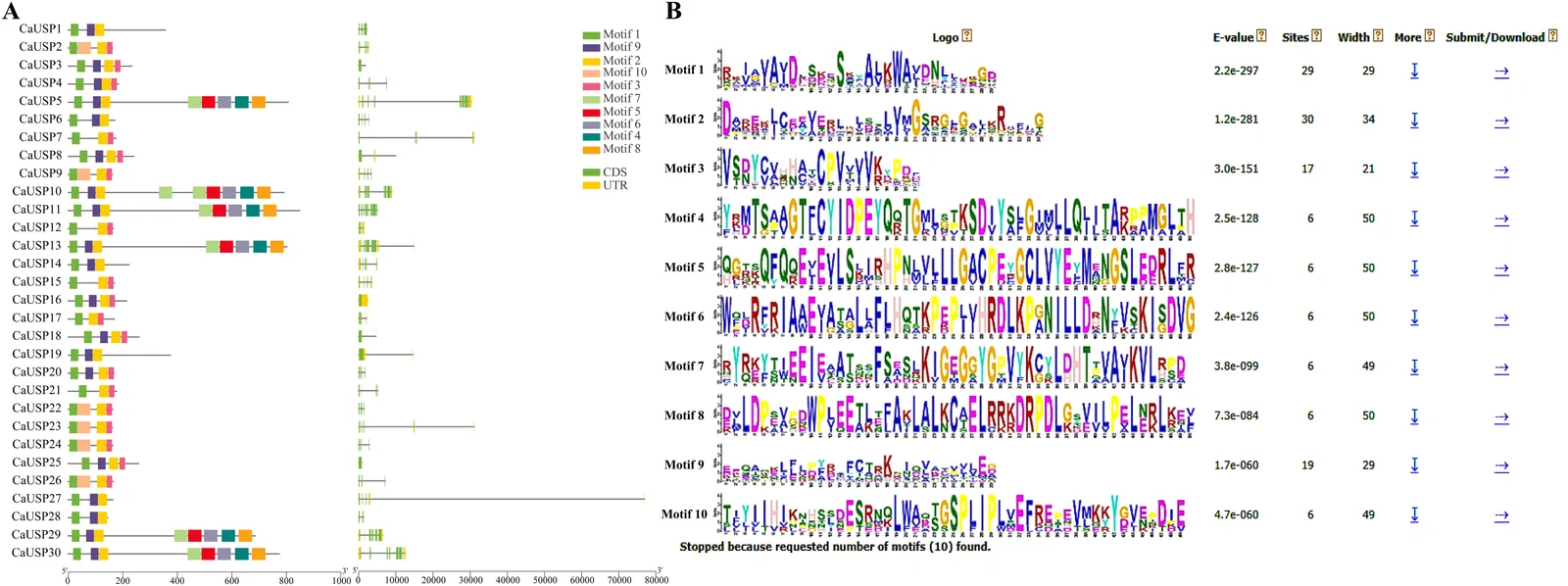
Conserved motifs and gene structure of CaUSP members. **(A)** Left panel: motif distribution among CaUSP members. Right panel: yellow boxes represent the untranslated 5′ and 3′ regions, green boxes represent exons, and black lines represent introns. **(B)** Ten conserved motif patterns of CaUSP members are shown.

### 2.4 Analysis of *cis*-acting elements in the CaUSP family

The 2-kb promoter regions upstream of the *CaUSP* genes were extracted, and *cis*-regulatory elements (CREs) were predicted using the online database PLANTCARE. In total, 32 types of CREs were identified. These elements could be classified into three major functional categories: (1) stress-related (10 types), including ARE, DRE-core, LTR, MBS, and MYB motifs; (2) hormone-responsive (10 types), such as ABRE, as-1, CARE, and CGTCA-motif; and (3) development-associated (12 types), covering A-box, AE-box, Bos-4, and CAT-box (**Figure 4**).

**Figure 4:**
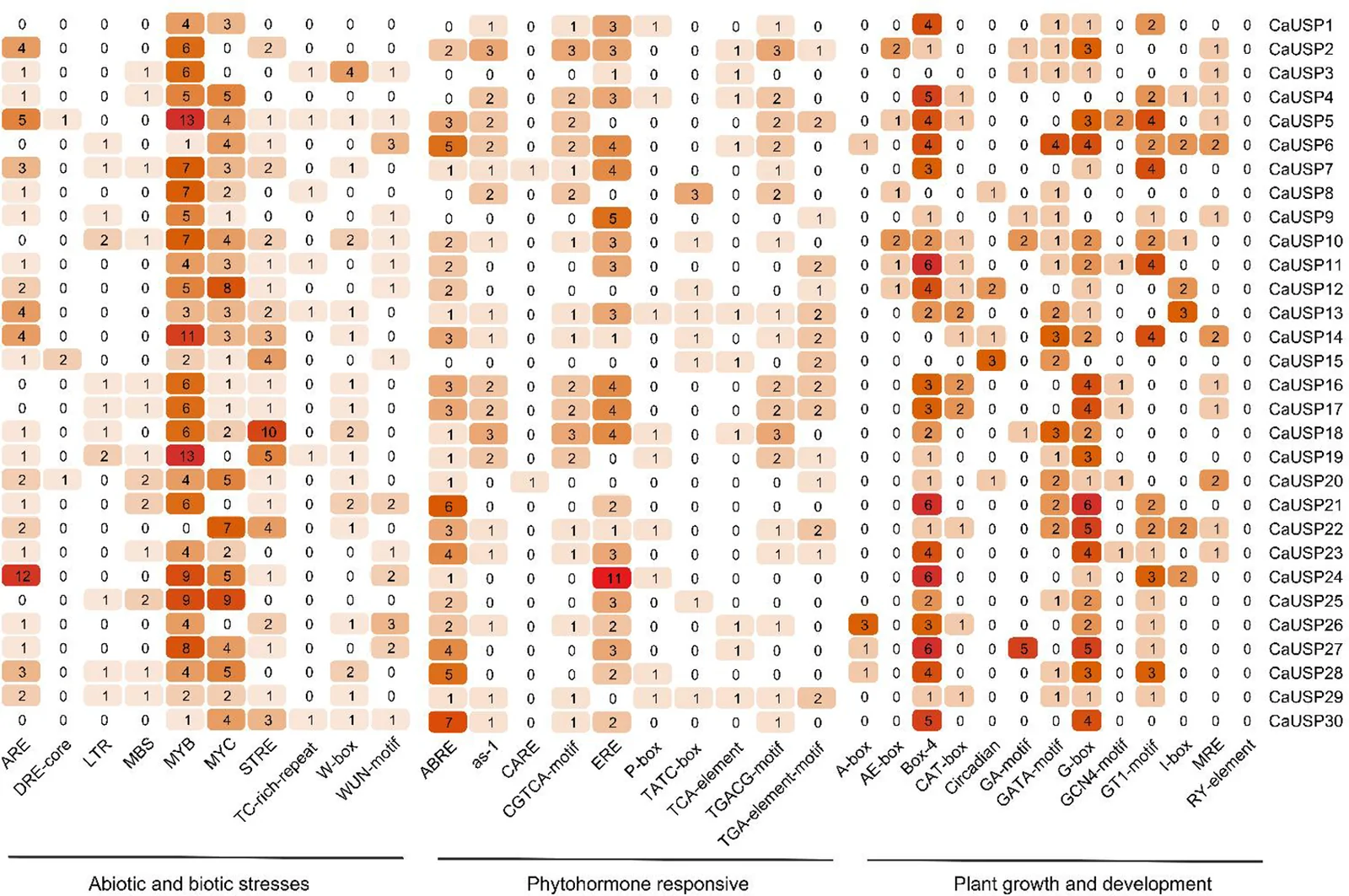
Analysis of cis-acting elements in the CaUSP Family, the numbers indicate the quantity of the elements.

### 2.5 Genome-wide collinearity analysis of CaUSP family members

To assess evolutionary conservation, we performed a genome-wide collinearity analysis of the *CaUSP* family between pepper and two representative species: rice (a monocot) and *Arabidopsis* (a eudicot). The analysis identified 13 and 16 collinear gene pairs between pepper and rice and between pepper and *Arabidopsis*, respectively (**Figure 5**). These syntenic relationships suggest that the *CaUSP* gene family is evolutionarily conserved and likely maintains consistent functions across divergent plant lineages.

**Figure 5:**
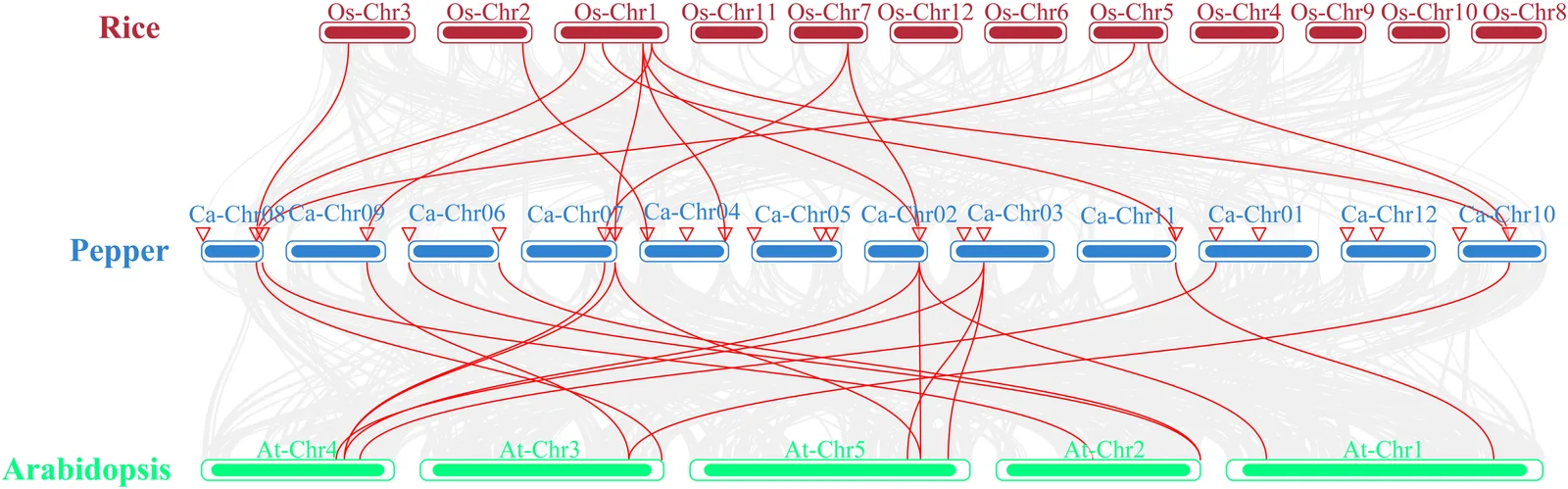
Evolutionary analyses of CaUSP gene family members. Gray lines in the background indicate the collinear blocks within *Arabidopsis*, rice and pepper, while red lines highlight the syntenic *CaUSP* gene pairs.

### 2.6 Expression patterns of CaUSP genes in flower

To explore the potential functions of *CaUSP* genes, we first analyzed their expression profiles across nine floral developmental stages using a public transcriptome database (PepperHub; http://lifenglab.hzau.edu.cn/PepperHub/) (Feng et al., 2017). Based on the FPKM values (**Figure 6A**), *CaUSP19* showed markedly higher transcript abundance compared to other family members, with low expression in early stages but sharp up-regulation in later stages (**Figure 6A**; **Supplementary Table S5**). We then performed quantitative real-time PCR (qPCR) to validate the expression patterns of all 30 *CaUSP* genes (**Figure 6B**; **Supplementary Figure S1**). The qPCR results confirmed that nine genes—*CaUSP19*, *CaUSP1*, *CaUSP4*, *CaUSP12*, *CaUSP10*, *CaUSP13*, *CaUSP14*, *CaUSP29*, and *CaUSP30*—were up-regulated during late floral development. Among these, *CaUSP19* showed the highest transcript levels from stages F7 to F9 (**Figure 6B**). Given its exceptionally high expression during late flower bud development (**Figure 6**; **Supplementary Table S5**), we selected *CaUSP19* for further functional analysis. This pronounced expression pattern implies a potentially important role for *CaUSP19* in floral development.

**Figure 6:**
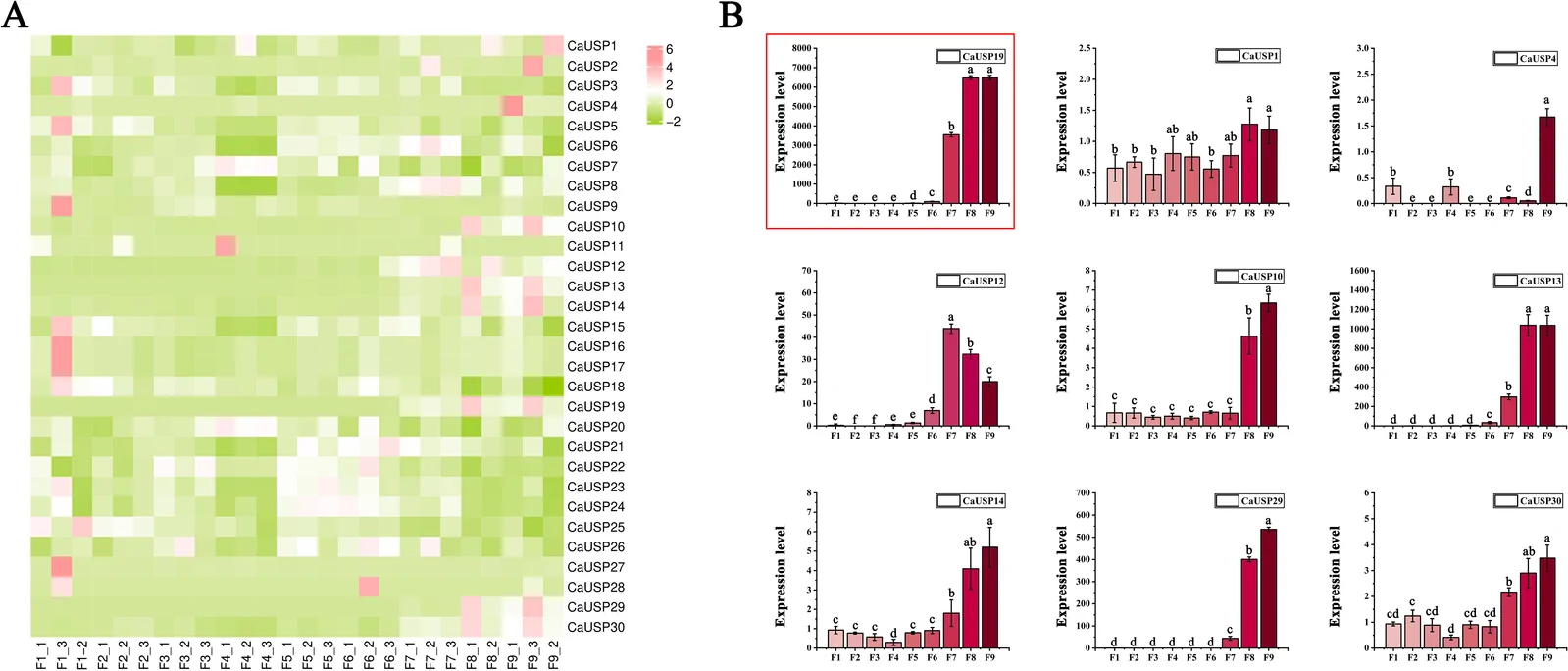
Expression pattern of *CaUSP* genes across 9 floral developmental stages in pepper. **(A)** Expression of 30 *USP* genes across nine developmental stages, derived from transcriptomic data in PepperHub. **(B)** Expression analysis of the 9 *USP* genes across the same nine developmental stages by qPCR. Different letters indicate significant differences (one-way ANOVA, Tukey’s test, *P* < 0.05).

### 2.7 Silencing CaUSP19 reduces pollen viability in pepper

To validate the role of *CaUSP19* in floral development, we employed virus-induced gene silencing (VIGS) to knock down its expression in pepper. Quantitative reverse-transcription PCR (qRT-PCR) confirmed that *CaUSP19* transcript levels were significantly reduced in silenced plants compared with controls (**Figure 7A**). Consistent with this molecular phenotype, potassium iodide staining of pollen collected at anthesis showed a substantial decrease in pollen viability upon *CaUSP19* silencing (**Figures 7B, D**). We further performed pollen germination assays on pollen from plants with silenced *CaUSP19*, and the results showed that the pollen germination rate was significantly reduced following *CaUSP19* silencing (**Figures 7C, E**). Together, these results demonstrate that *CaUSP19* is essential for normal pollen development and function in pepper.

**Figure 7:**
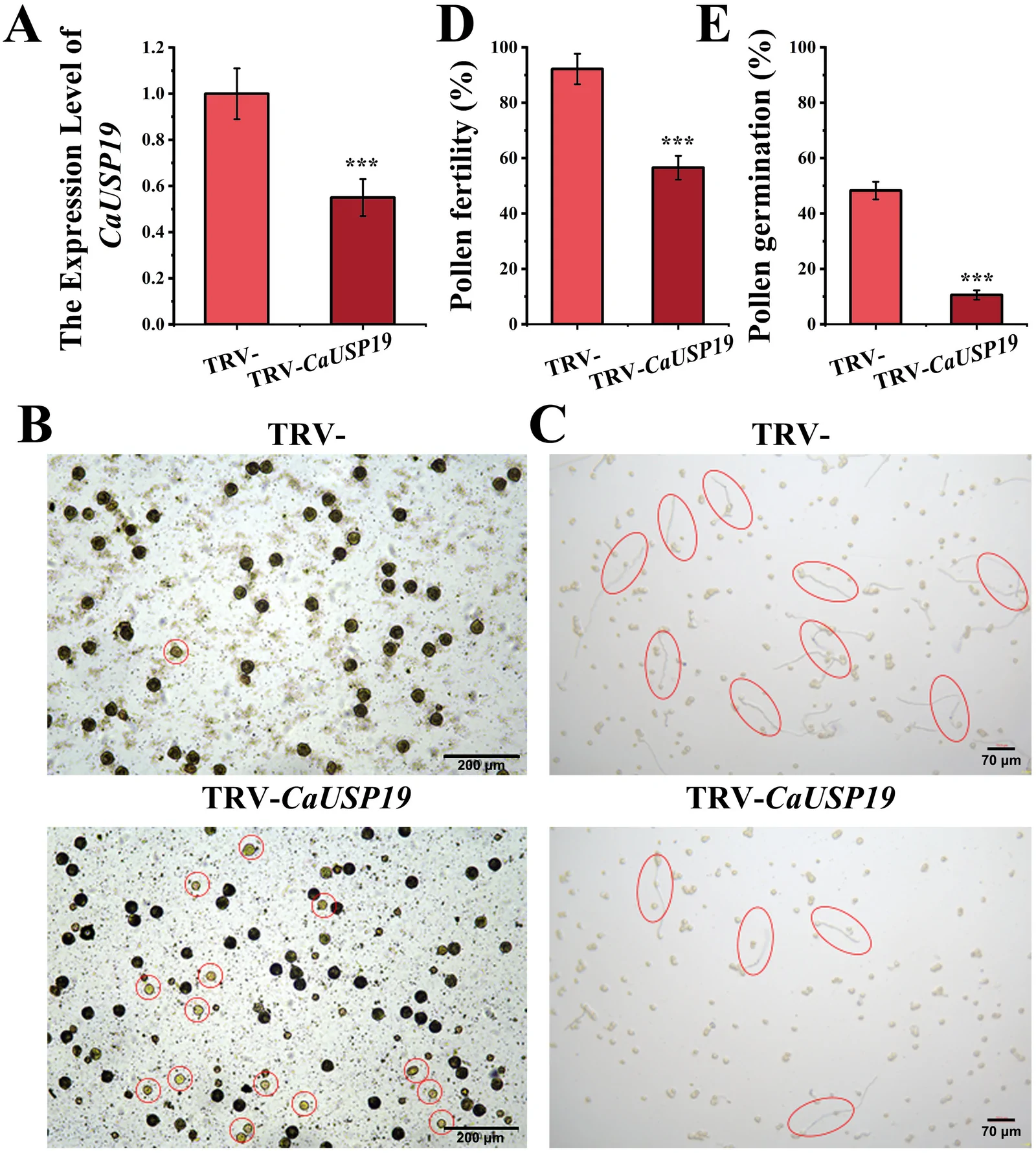
Reduced pollen viability and germination rates upon *CaUSP19* silencing. **(A)** *CaUSP19* expression in silenced plants. **(B)** Representative images of KI/I_2_-stained pollen grains from control (TRV-) and *CaUSP19*-silenced (TRV-*CaUSP19*) plants. Red circles indicate representative non-viable (unstained/aborted) pollen grains. **(C)** *CaUSP19* silencing impairs pollen germination in pepper. Red circles indicate representative germinated pollen grains. **(D)** Statistical analysis of pollen viability (percentage of viable pollen grains). **(E)** Statistical analysis of pollen germination rates. (n = 3 biological replicates, with at least 300 pollen grains counted per replicate). RT-qPCR analyses were performed with three biological replicates. Asterisks indicate significant differences by Student’s *t*-test (*P* < 0.001***).

## 3 Discussion

The USP family comprises stress-responsive proteins conserved across diverse organisms. Although USP genes have been characterized in several plants—including *Arabidopsis*, tobacco, rice, and potato (Margret et al., 2002; Silvia et al., 2015; Pushpika et al., 2016; Tianshuai et al., 2024)—a systematic investigation of this family in the economically important solanaceous crop pepper (*Capsicum annuum* L.) was absent. Here, we identified 30 *CaUSP* genes in the pepper genome and performed a comprehensive analysis of their phylogeny, expression, and function, thereby establishing a foundation for understanding USP roles in pepper growth, development, and environmental adaptation.

Phylogenetic analysis grouped the 30 *CaUSP* genes into eight distinct subgroups (**Figure 2**), reflecting possible evolutionary and functional divergence. Collinearity analysis revealed 16 syntenic pairs between pepper and *Arabidopsis* and 13 between pepper and rice, supporting the ancient origin of this gene family. *Cis*-acting element screening revealed that *CaUSP* promoters are enriched with motifs responsive to light, abscisic acid (ABA), jasmonic acid (JA), and drought (e.g., ABRE, CGTCA-motif, ARE, DRE-core). This is consistent with previous reports that USPs in *Arabidopsis* and rice are induced by salinity, dehydration, and oxidative stress. The presence of multiple hormone-related elements suggests that *CaUSP* genes may integrate stress and developmental signals. For example, *CaUSP19* contains both ABRE (ABA-responsive) and CGTCA-motif (JA-responsive) elements in its promoter. While ABA and JA are known to play important roles in pollen development and fertility, the presence of these motifs is not unique to *CaUSP19*. Therefore, our observation that *CaUSP19* may act downstream of these phytohormones in influencing pollen viability remains a hypothesis that requires future experimental validation through hormone-treatment time courses and promoter-reporter assays.

Consistent with the well-established involvement of USPs in stress responses (Hunseung et al., 2012; Silvia et al., 2015; Young Jun et al., 2016; Sarah Mae Boyles et al., 2017), we found that promoters of several *CaUSP* genes contain stress-related elements such as ARE, DRE-core, and MYB, implying a broad role for this family in abiotic stress adaptation in pepper, as similarly revealed by integrated promoter and expression analyses of heat-shock protein families in tropical crops (Alam et al., 2024). One of the most striking findings of this study is the stage-specific expression of *CaUSP19* during late-stage flower development and its positive association with pollen viability. Most previous studies focused on USPs as stress-inducible proteins involved in cellular homeostasis and energy management under adverse conditions. Plant USPs show diverse domain structures and function both in stress responses and development: they act as chaperones (*Arabidopsis*), mediate osmotic/ABA signaling (tobacco, grapevine), and are widely induced by drought, UV-B, or exogenous ABA across multiple species (Margret et al., 2002; Maqbool et al., 2009; Hunseung et al., 2012; Silvia et al., 2015; Pushpika et al., 2016; Young Jun et al., 2016; Sarah Mae Boyles et al., 2017; Xiaoyue et al., 2022; Jian et al., 2023; Yan et al., 2023; Tianshuai et al., 2024; Ruiqiang et al., 2025); additionally, specific USPs regulate seed germination, circadian rhythms, flowering (Arabidopsis, GRUSP), and starch accumulation in sweet potato (Gorshkova et al., 2018; Kieu Anh Thi et al., 2022; Jian et al., 2023).

Notably, *CaUSP19* displayed a stage-specific and strongly up-regulated expression pattern during late bud development (**Figure 6**), pointing to a potential function in floral organ formation. Functional validation using VIGS-mediated silencing confirmed that knockdown of *CaUSP19* significantly impairs pollen viability (**Figure 7**), establishing *CaUSP19* as a key regulator of male reproductive development in pepper, extending the functional scope of *USP* genes beyond classical stress responses.

In summary, this study provides the first genome-wide identification and characterization of the USP gene family in pepper, and uncovers a previously unknown role for *CaUSP19* in controlling pollen viability. These findings expand the functional repertoire of plant USPs canonical stress adaptation and establish a critical link between stress-related USP genes and reproductive development. Moreover, the stage-specific expression and promoter architecture of *CaUSP19* suggest potential crosstalk between hormonal signaling and floral development, offering a fresh perspective on the multifunctional nature of USP proteins. Note that the subcellular localization analysis was entirely based on bioinformatic predictions, and transient expression verification of CaUSP19 is warranted in future work. Future work should focus on generating stable mutant lines to definitively establish the causal role of *CaUSP19* in pollen fertility, as well as investigating the functions of other *CaUSP* members in both floral development and abiotic stress tolerance. Additionally, exploring the natural variation of *CaUSP* genes across pepper germplasm may facilitate their breeding utility for improving abiotic resilience and reproductive stability in this economically important crop.

## 4 Materials and methods

### 4.1 Plant materials and growth conditions

For the VIGS experiments, the cultivar ‘S8’ (*Capsicum annuum*) was used. All plants were cultivated in a greenhouse at the Hunan Academy of Agricultural Sciences (Changsha, China) under strictly controlled conditions: a 16-h photoperiod at 24 °C/an 8-h dark period at 19 °C, with the relative humidity maintained at 65%.

### 4.2 Identification of USP genes

The pepper genome sequence and annotation files were retrieved from PepperBase (http://www.bioinformaticslab.cn/PepperBase/download/). The reference genome was derived from the *Capsicum annuum* accession ‘Zunla-1’. The published pepper genome assembly of the cultivar ‘Zunla-1’ (Zunla-1_v3.0, BioProject accession no. PRJCA025503), retrieved from PepperBase. Putative USP genes were initially screened by homology search against the NCBI RefSeq database using BLASTP. Candidate coding sequences (CDS) and their corresponding protein sequences were then extracted using TBtools (Chen et al., 2023). To validate conserved domains, candidate protein sequences were analyzed with HMMER 3.0 against the SMART database, using an E-value threshold of 0.01. Redundant sequences were manually removed. A subsequent BLAST conserved domain search was performed to confirm the presence of the target USP domain. Finally, the molecular weight and theoretical isoelectric point (pI) of each non-redundant protein were predicted using the ExPASy ProtParam online tool (https://web.expasy.org/protparam/).

### 4.3 Chromosomal location, duplication, and homology analysis of USP genes

Based on the PepperBase genome assembly, the chromosomal locations of *CaUSP* genes were mapped. Gene duplication events within the pepper genome were investigated, and a duplication map was generated to illustrate tandem and segmental duplication patterns. To examine evolutionary conservation, we performed a comparative synteny analysis between pepper and two reference species, *Arabidopsis thaliana* and *Oryza sativa*. Genome and annotation files for both species were retrieved from public databases. Synteny diagrams were constructed and visualized using TBtools (Chen et al., 2023).

### 4.4 Phylogenetic analysis and classification of USP proteins

To reconstruct the evolutionary relationships of USP proteins, sequences from *Arabidopsis thaliana*, *Oryza sativa*, and *Capsicum annuum* were aligned using MEGA software (Sudhir et al., 2016). Evolutionary distances were estimated under the Poisson model, and gaps were handled with the pairwise deletion method. A phylogenetic tree was then constructed using the neighbor-joining (NJ) method (Sudhir et al., 2016). The resulting topology was visualized and refined using FigTree v1.4.3, and final graphical adjustments were made in Adobe Illustrator CC 2019 for clarity and presentation.

### 4.5 Analysis of Cis-Regulatory Elements in CaUSP Gene Promoters

To identify putative *cis*-regulatory elements (CREs), genomic DNA sequences spanning 2 kb upstream of the transcription start site of each *CaUSP* gene were extracted (Chen et al., 2023). This promoter length was chosen in accordance with common practice in plant promoter analysis to ensure coverage of major regulatory motifs. The extracted sequences were submitted to the PlantCARE online database for *in silico* prediction of CREs. Using PlantCARE (Magali et al., 2002), the type, precise location, and copy number of each predicted *cis*-acting element were determined. The putative regulatory functions of these elements were inferred based on their previously annotated roles in plant hormone response, stress signaling, or development.

### 4.6 Analysis of USP protein structure, conserved motifs, gene structure, and phylogenetic tree

Gene structures (exon–intron organizations) of the identified *CaUSP* genes were extracted from the genome annotation and visualized using TBtools. Conserved protein motifs were predicted with the online tool MEME v5.1.1 (http://meme-suite.org/tools/meme) under default parameters (Bailey et al., 2006). Phylogenetic analysis was conducted as described above.

### 4.7 Virus-induced gene silencing experiment

A virus-induced gene silencing (VIGS) vector targeting *CaUSP19* was constructed. A 300-bp gene-specific fragment corresponding to nucleotides 524–823 of the CaUSP19 coding sequence (CDS), designed using the SGN VIGS tool (https://vigs.solgenomics.net/), was cloned into the TRV2 vector. The resulting recombinant plasmid (TRV2-*CaUSP19*) was introduced into *Agrobacterium tumefaciens* strain GV3101 by electroporation. For agroinfiltration, plants were separated into two treatment groups: empty-vector control (TRV2-), and *CaUSP19*-silenced (TRV2-*CaUSP19*). Agroinfiltration was carried out following an established protocol (Yi et al., 2022).

### 4.8 Quantitative real-time polymerase chain reaction

Total RNA was isolated from all samples using TransZol reagent (TransGen Biotech, Beijing, China), with three biological replicates per treatment group. First-strand cDNA was synthesized from the extracted RNA using HiScript^®^ II Q RT SuperMix (Vazyme, Nanjing, China). Quantitative real-time PCR (qRT-PCR) was then performed on a LightCycler^®^ 96 system (Roche) with ChamQ Universal SYBR qPCR Master Mix (Vazyme). The pepper *actin* gene (*CaActin*) was used as the internal control for normalization. Gene expression levels were normalized using the 2−ΔΔCT method (Schmittgen and Livak, 2008).

### 4.9 Iodide staining assay

Pollen viability was assessed using potassium iodide–iodine (KI/I_2_) staining. Fresh pollen grains were collected from newly opened flowers and incubated in I_2_-KI solution for 10 min at room temperature. After rinsing with distilled water, stained pollen grains were immediately observed under a light microscope. Viable pollen grains stained dark blue, while non-viable (aborted) pollen grains remained unstained or light yellow. At least 300 pollen grains were counted per replicate, and the percentage of viable pollen grains was calculated. The staining procedure followed a previously described protocol (Wang et al., 2015).

### 4.10 Pollen germination assay

Pollen germination was evaluated using an *in vitro* liquid culture method. Pollen grains were collected from freshly opened flowers of *Capsicum annuum* L. and suspended in a germination medium containing 10% sucrose, 0.1 mM H_3_BO_3_, and 1 mM CaCl_2_. The suspension was spread on glass slides and incubated at 25 °C in a humid dark chamber for 3–4 h. A pollen grain was considered germinated when the pollen tube length exceeded the grain diameter (Mercado et al., 1994).

## 5 Conclusions

We systematically identified 30 *CaUSP* genes in pepper and demonstrated that *CaUSP19* is associated with pollen fertility by affecting pollen viability. These findings provide a foundation for further functional studies of USP family members in pepper reproduction.

## Data Availability

The datasets presented in this study can be found in online repositories. The names of the repository/repositories and accession number(s) can be found in the article/**supplementary material**.
